# Routine measurement of outcomes in Australia's public sector mental health services

**DOI:** 10.1186/1743-8462-2-8

**Published:** 2005-04-19

**Authors:** Jane Pirkis, Philip Burgess, Tim Coombs, Adam Clarke, David Jones-Ellis, Rosemary Dickson

**Affiliations:** 1 Program Evaluation Unit, School of Population Health, The University of Melbourne, Melbourne, Australia; 2 Queensland Centre for Mental Health Research, The University of Queensland, Brisbane, Australia; 3 New South Wales Institute of Psychiatry, Sydney, Australia; 4 Strategic Data Pty Ltd, Melbourne, Australia

**Keywords:** Mental health, outcomes, routine outcome measurement, Australia

## Abstract

**Objective:**

This paper describes the Australian experience to date with a national 'roll out' of routine outcome measurement in public sector mental health services.

**Methods:**

Consultations were held with 123 stakeholders representing a range of roles.

**Results:**

Australia has made an impressive start to nationally implementing routine outcome measurement in mental health services, although it still has a long way to go. All States/Territories have established data collection systems, although some are more streamlined than others. Significant numbers of clinicians and managers have been trained in the use of routine outcome measures, and thought is now being given to ongoing training strategies. Outcome measurement is now occurring 'on the ground'; all States/Territories will be reporting data for 2003–04, and a number have been doing so for several years. Having said this, there is considerable variability regarding data coverage, completeness and compliance. Some States/Territories have gone to considerable lengths to 'embed' outcome measurement in day-to-day practice. To date, reporting of outcome data has largely been limited to reports profiling individual consumers and/or aggregate reports that focus on compliance and data quality issues, although a few States/Territories have begun to turn their attention to producing aggregate reports of consumers by clinician, team or service.

**Conclusion:**

Routine outcome measurement is possible if it is supported by a co-ordinated, strategic approach and strong leadership, and there is commitment from clinicians and managers. The Australian experience can provide lessons for other countries.

## Introduction

Internationally, there is an increasing emphasis on routine outcome measurement in mental health. A push to improve quality of care for consumers has prompted interest in monitoring outcomes at an individual level, and financial pressures and a need to demonstrate value-for-money have led to the use of aggregate reports that allow comparisons between services [1]. In the United States, there are examples of routine outcome measurement being 'rolled-out' across mental health services in entire states, such as the Ohio Mental Health Consumer Outcomes System [2]. In Europe, there are also some examples of individual services monitoring outcomes, as in the South Verona Outcomes Project [3] and the MECCA Study [4], but the routine collection of outcome data has not extended to larger areas.

Australia's commitment to routine outcome measurement is evidenced in its National Mental Health Strategy [5-7]. Since its inception in 1992, the continued improvement of the quality and effectiveness of the treatment of people with a mental illness has been a key objective of the Strategy. The Strategy has recognised that this objective can only be achieved through the development of sound information to support service planning and delivery, and consequently the systematic implementation of routine outcome measurement in all public sector mental health services is one of its priorities.

State/Territory governments and the Australian Government are collaborating in a coherent national approach. For their part, all States/Territories have signed Agreements that require them to routinely submit two sets of data from public sector mental health services to the Australian Government. Firstly, they are required to submit de-identified, patient-level outcome data, referred to as the 'National Outcomes and Casemix Collection' (NOCC) [8]. These outcome data are collected via a range of instruments that incorporate clinician and consumer perspectives on a range of mental health related constructs (e.g., symptomatology, level of functioning, degree of disability) relevant to adults, children/adolescents and older people. The idea is that administration of these instruments at specific points in time will allow services to monitor changes in individual consumers and in groups of consumers, and, ultimately, to make comparisons with similar consumers in like services. Table 1 shows the specific instruments that comprise the NOCC dataset.

**Table 1:** Data comprising the NOCC collection

		**Adults**	**Older persons**	**Children and adolescents**
**Clinician-rated**	Principal and additional diagnoses	√	√	√
	Mental health legal status	√	√	√
	Health of the Nation Outcomes Scale (HoNOS) [16]	√		
	Health of the Nation Outcomes Scale 65+ (HoNOS65+) [17]		√	
	Health of the Nation Outcomes Scale for Children and Adolescents (HoNOSCA) [18]			√
	Life Skills Profile 16 (LSP-16) [19, 20]	√	√	
	Resource Utilisation Groups – Activities of Daily Living Scale (RUG-ADL) [21]		√	
	Focus of Care [20]	√	√	
	Children's Global Assessment Scale (CGAS) [22]			√
	Factors Influencing Health Status (FIHS) [20]			√
**Consumer-rated**	Mental Health Inventory (MHI) [23] or Behaviour and Symptom Identification Scale (BASIS-32) [24] or Kessler-10 Plus (K-10+) [25]	√	√	
**Consumer- and parent-rated**	Strengths and Difficulties Questionnaire (SDQ)[26]			√

Source: Department of Health and Ageing (2003) [27]

Secondly, States/Territories are required to submit data on inpatient episodes of care and community contacts, termed the 'National Minimum Data Set – Mental Health Care' (NMDS) [9-11]. These data provide information on resource use by consumers, and, when combined with the above outcome data will promote the development and informed use of casemix to understand the role of provider variation in differences between agencies' costs and outcomes. For the purposes of the current paper, however, the focus is routine outcome measurement only, rather than casemix development.

For its part, the Australian Government has established three Expert Groups (Adult, Child/Adolescent, and Older Persons) to advise on the implementation and use of routine outcome data in mental health services. It has also provided resources to support training in the use of outcome measures, and arrangements to receive, process, analyse and report on outcome data submitted by States/Territories.

The latter arrangements have been established through the 'Australian Mental Health Outcomes and Classification Network' (AMHOCN), a consortium contracted from late 2003 to provide national leadership in the development of outcome measurement in mental health. AMHOCN is pursuing a work program with three components, each being undertaken by a different member of the consortium: data management (Strategic Data Pty Ltd, Victoria); analysis and reporting (Queensland Centre for Mental Health Services Research, The University of Queensland, Queensland); and training and service development (New South Wales Institute of Psychiatry, New South Wales).

An immediate concern for AMHOCN was determining States/Territories' progress with respect to 'rolling out' routine outcome measurement, so each State/Territory was invited to participate in a consultation with AMHOCN. This paper reports on the findings from these consultations.

## Method

The consultations occurred in March/April 2004. The intention was to seek a range of views, rather than to try to achieve a representative sample, and States/Territories were asked to nominate relevant stakeholders. They could choose whomever they wished, but they were advised to consider including policy-makers and technical personnel from central mental health units and mainstream health information sections, as well as consumers/carers. Most States/Territories sent representatives from all of these groups, and many also sent service managers, clinicians, individuals responsible for supporting routine outcome measurement at a site level, and members of the Expert Groups. In total, 123 individuals attended the consultations: 10 from New South Wales; 21 from Victoria; 28 from Queensland; 23 from Western Australia; 17 from South Australia; six from both Tasmania and the Australian Capital Territory; and 12 from the Northern Territory. Many 'wore several hats', rendering it difficult to provide a breakdown of their roles.

The consultations sought answers to questions regarding progress in four domains: (a) data collection systems and infrastructure; (b) training and retraining of staff; (c) the implementation of routine outcome measurement; and (d) and analysis, reporting and use of data.

The majority of consultations took place over a full day, with the shortest being half a day. All of the consultations began with a brief presentation from AMHOCN, and then elicited information from participants. Some States/Territories chose to split the consultation in two, inviting policy makers, planners and clinicians to attend one session and technical personnel to attend the other. Some States/Territories gave formal presentations responding to specific questions; others took a more informal approach. In some cases, the information presented at the consultations was supplemented by a written response.

Each consultation was transcribed. The transcription was combined with any other written material (e.g., formal responses and presentations), and examined at a global level to identify major themes within each domain. Individual responses were classified according to these themes. Each State/Territory was given the opportunity to comment on the accuracy of the written interpretation of the consultation.

## Results

### States/Territories' progress regarding data collection systems and infrastructure

Ultimately, States/Territories are aiming to have streamlined data collection systems that allow the outcome data collected via the NOCC dataset to be linked to the admitted and non-admitted activity data in the NMDS. This will allow outcome data to be 'attached' to given inpatient and community episodes of care. This has advantages in terms of allowing outcomes for consumers to be 'tracked' across episodes, and is necessary for progressing casemix development work that requires outcome data and resource use data to be combined within episodes.

All States/Territories have developed data collection systems, or are in the final stages of doing so. For some, this has involved 'starting from scratch'; for others it has required modifications to existing systems. For example, the systems used in Queensland to capture admitted and non-admitted NMDS information did not have the functionality to incorporate outcome measures, so an additional system was developed to do so. By contrast, in the Australian Capital Territory, the system used by all community teams to collect non-admitted NMDS data, was modified to collect outcome data and extended to inpatient services, where it runs alongside a separate patient administration system for the collection of inpatient activity data.

States/Territories differ in terms of the number of systems that are currently involved in the collection of routine outcome data. The simplest scenario is one where outcome measurement functionality has been added to an existing system for recording activity in community mental health settings, and has been extended into inpatient settings (as with the system in the Australian Capital Territory, described above). This also occurs in Victoria, Tasmania and the Northern Territory. Other States rely on as many as four statewide systems to collect NOCC and NMDS information, sometimes with further degrees of complexity between areas or metropolitan/country settings.

Linking NOCC and admitted and non-admitted NMDS datasets is impeded in most States/Territories by the lack of a unique identifier. Typically, linkage is only possible for parts of the data (usually NOCC and non-admitted NMDS data) and/or by conducting quite complex record linkage tasks. The exception is the Northern Territory, which has a client master index that allocates each consumer a unique identifier that allows him/her to be 'tracked' across episodes, across services, and over time. Other States/Territories are working towards improvements, but have some way to go. Western Australia's data collection system has a unique identifier that will allow episodes of care to be attributed to the same individual, regardless of location or time, but its 'roll-out' is not yet completed. Queensland and New South Wales have plans to reconcile their unique identifier systems via specific projects. This will mean that States will assign a unique identifier to a given individual that he or she will 'carry' across all health services, including mental health services, but this will not occur in the near future.

States/Territories have differing levels of infrastructure to support the NOCC and NMDS collections. Human resources vary, with some States/Territories having a number of personnel deployed to train and support clinicians and managers, and others relying on one or two core individuals. So, for example, Queensland has Zonal Outcomes Co-ordinators and Mental Health Information Support Officers providing 'on the ground' support, whereas Tasmania has a small, centrally-located team performing the same function. Physical resources also vary, with some States/Territories having sophisticated online data entry systems (e.g., the Australian Capital Territory), others relying on batch entry of paper-based forms (e.g., Tasmania), and still others using a combination of the two (e.g., New South Wales and South Australia).

### States/Territories' progress regarding training and retraining of staff

All States/Territories have implemented comprehensive training programs and have trained substantial proportions of their mental health workforces in routine outcome measurement. According to stakeholders, well over 7,000 clinicians and managers across Australia have received direct training, and possibly half as many again have received training under a train-the-trainer model. This figure is consistent with that of 10,000 reported by the Department of Health and Ageing, which is estimated to represent approximately 60% the public sector mental health workforce [12].

The direct training approach is seen as having the benefit of consistency, while the train-the-trainer approach is seen as fostering capacity building and being less labour intensive and cheaper. Some States/Territories have considered accrediting trainers, so that the advantages of both approaches can be combined. Managers are also more commonly being recruited as trainers, as part of a move to secure their commitment in leading the change process. South Australia has been innovative here, building capacity by training staff as trainers through the Certificate 4 in Workplace Training and Assessment, and investing in training in content knowledge around outcome measures in the NOCC collection. In this way, South Australia has addressed some of the difficulties inherent in more standard train-the-trainer approaches.

Many States/Territories are now beginning to consider issues of ongoing training and support. High levels of staff turnover in some States/Territories mean that there are new staff who have not been trained, and lags between training and implementation in some jurisdictions have resulted in skills being lost. In addition, many States/Territories are recognising the need for a second wave of training that goes beyond how to use the outcome measures and focuses more on how to interpret the results of specific measures (at individual and aggregate levels).

Some States/Territories have implemented ongoing training strategies. Western Australia, for example, has begun refresher training. Tasmania has implemented a second round of training, focusing on the outcome measures that were not covered in the original training (i.e., the LSP-16 and the BASIS-32). Queensland has established an ongoing training program that emphasises sustainability, clinical utility and building capacity, and involves its Zonal Outcomes Co-ordinators modelling for clinicians how outcome data can be used in clinical management. Most other jurisdictions have plans in place to implement a second wave of training that focuses on the clinical and management utility of outcome measurement.

Novel, clinician-focused approaches, such as the use of vignettes and interactive case studies in Victoria and Western Australia, have underpinned the initial and ongoing training in many States/Territories. Training has also typically involved the development of resources (e.g., guides and glossaries for specific measures, consumer/carer brochures), many of which are located on individual State/Territory websites.

### States/Territories' progress regarding the implementation of routine outcome measurement

States/Territories are now implementing routine outcome measurement, albeit with very variable degrees of progress. By May 2004, Victoria had provided data for 2000–01, 2001–02 and 2002–03; New South Wales for 2001–02 and 2002–03; Tasmania for 2001–02; and Western Australia, Queensland and the Northern Territory for 2002–03 (partial year only in the latter two). For South Australia and the Australian Capital Territory, 2003–04 data will constitute the first report. Within States/Territories, there is considerable patchiness in terms of coverage, compliance and completeness. There is variability by setting (with community services generally having higher coverage than inpatient services) and by outcome measure (with clinician-rated measures being completed to a greater extent than consumer-rated measures). Strong leadership at all levels has been associated with high levels of overall performance in terms of implementation.

Beyond initial training and rollout, some States/Territories have considered how to sustain and build upon current efforts with regard to routine outcome measurement. There is recognition by these (and other) States/Territories that unless routine outcome measurement becomes embedded in the process of clinical care, it will not be seen as a priority by clinicians and managers. So, for example, in New South Wales outcome measurement has been embedded in a standard protocol, which involves triage, assessment, review and discharge documentation. Specifically, a suite of clinical modules has been developed that not only includes an outcomes module but also includes the incorporation of outcome measures into the process of care. For example, the collaborative care planning module encourages collaboration between the clinician and consumer, and prompts review of the clinician-rated HoNOS and the consumer-rated K-10. The process of embedding outcome measurement within the clinical process of care is enhanced by providing clinical interpretations of given scores on particular measures. All New South Wales Area Mental Health Services will have the same modules, produced as standard medical record stationery for use within clinical files.

### States/Territories' progress regarding analysis and reporting of data

Some States/Territories have also begun to consider how best to provide feedback to staff. There is recognition that without appropriate and timely feedback in the form of relevant reports that shed light on clinical and management issues, the current momentum will falter and data quality and comprehensiveness will be jeopardised. Feedback in the form of reports is required at a variety of levels. Some States/Territories have developed individual-level reports that allow clinicians to profile an individual consumer's scores on a range of outcome measures, either at a single point in time or over time. For example, in the Australian Capital Territory, the data capture system produces an electronic management plan, similar to the New South Wales module described above, which incorporates areas that the clinician and consumer might want to address, given the consumer's profile on the outcome measures. Similarly, in Western Australia, HoNOS scores of greater than 2 on Items 1 (Overactive, aggressive, disruptive or agitated behaviour) and 2 (Non-accidental self injury) trigger a risk assessment, and an alert is registered on the system.

Other States/Territories are generating aggregate-level reports about compliance. For instance, Western Australia generates Statewide compliance reports that are distributed to mental health services every six weeks, and the Office of Mental Health works with services that are experiencing difficulties with compliance to review the systems in place for monitoring the NOCC collection.

A few States/Territories have started producing some rudimentary, aggregate-level reports that provide information about groups of consumers under the care of a given clinician, team or service. Tasmania, for example, has produced monthly reports for its Southern Region, which include aggregate-level data on average HoNOS scores at admission, review and discharge. Some States/Territories have begun to consider how best to provide these reports to areas and services. New South Wales, for example, has conducted a project involving workshops in all area health services, using their own data to demonstrate the clinical and management utility of the information. A similar process has been undertaken in Queensland.

A range of factors has hampered efforts at analysis and reporting to date. These include resource issues (e.g., lack of personnel and technological constraints), data quality, a lack of clarity about which reports will have greatest clinical and management utility, and the absence of relevant normative and/or benchmarking data.

## Discussion

### Summary of findings

Australia has made an impressive start to nationally implementing routine outcome measurement in mental health services, although it still has a long way to go. All States/Territories have established data collection systems, although some are more streamlined than others. Significant numbers of clinicians and managers have been trained in the use of routine outcome measures, and thought is now being given to ongoing training strategies. Outcome measurement is now occurring 'on the ground'; all States/Territories will be reporting data for 2003–04, and a number have been doing so for several years. Having said this, there is considerable variability regarding data coverage, completeness and compliance. Some States/Territories have gone to considerable lengths to 'embed' outcome measurement in day-to-day practice. To date, reporting of outcome data has largely been limited to reports profiling individual consumers and/or aggregate reports that focus on compliance and data quality issues, although a few States/Territories have begun to turn their attention to producing aggregate reports of consumers by clinician, team or service.

### Study limitations

Several limitations must be borne in mind in interpreting the above findings. Firstly, the study was dependent upon States/Territories selecting the most appropriate stakeholders to attend the consultations. Guidance was provided, but it is possible that States/Territories were more inclined to invite those who were in favour of routine outcome measurement, and that the views of some key stakeholders were missed. In particular, the perspective of 'coalface' clinicians was not well captured. Anecdotal reports suggest that there is some apathy, cynicism and resistance towards outcome measurement among this group. Secondly, the study relied almost exclusively on subjective reports from the stakeholders who were present at the consultations. Standard qualitative methodologies were used to record and analyse their responses, but there were few opportunities for their views to be checked against any objective measures. Finally, routine outcome measurement is moving at a considerable pace in Australia, and further progress has been made since the time of the study. The study therefore provides a conservative picture of the status quo.

### Interpretation of findings

These limitations aside, some key messages emerge from the study. Specifically, it shows that routine outcome measurement is possible if it is supported by a co-ordinated, strategic approach and strong leadership. Equally important is commitment from clinicians who are involved in the day-to-day collection of the outcome data, and from managers who must make it a priority within their services. Stakeholders in the current study repeatedly stressed that this commitment will only be sustained in the long term if clinicians and managers value routine outcome measurement. Feedback to these groups in the form of reports tailored to their specific needs is crucial, and has been identified by others as necessary for maintaining momentum [2-4,13,14].

AMHOCN clearly has a role in taking routine outcome measurement to its next level. As a priority, AMHOCN is specifying a reporting framework for providing feedback to States/Territories. This involves considerations of the nature and form of data that AMHOCN itself will provide, as well as guidance to States/Territories about their own reporting. Several principles are guiding this process, in an effort to ensure that feedback has maximum clinical and management utility, and occurs as quickly as possible. Specifically, feedback should take the form of reports that are relevant and useful at a range of levels (e.g., individual, team, service and State/Territory). The precise nature of the reports should be informed by an iterative process, where relevant recipients are given the opportunity to comment on reports, and subsequent reports are modified accordingly. Reports should provide reference points that allow individual scores to be compared with normative data, and service profiles to be benchmarked against those of their peers. For now, reports will be based on NOCC data alone, in recognition of the difficulties in linking NOCC and NMDS data. This has implications for defining episodes of care, but provides scope for much to be done regarding reporting outcome data in a manner that is useful for clinicians and managers.

AMHOCN is also helping to consolidate the existing State/Territory training efforts. Specifically, it is working towards: developing and disseminating resources that fill particular gaps; helping States/Territories to streamline their training and re-training packages in a way that balances national consistency against the unique requirements of the local context; fostering the skills and knowledge required for interpreting and reflecting upon the meaning of outcome data, at a range of levels; encouraging information-sharing across the board, taking advantage of its 'birds eye view' to identify good ideas and approaches in given States/Territories and promote them in others; exploring processes for accrediting trainers, ensuring that national accreditation is consistent with and complementary to any existing accreditation efforts; and engaging, nurturing and supporting clinical leaders, champions and innovators.

## Conclusion

Australia has consistently been regarded as a world leader in routine mental health outcome measurement [15]. It is acknowledged that Australia still has some way to go before routine outcome measurement is 'bedded down', and issues of data coverage, completeness and compliance are fully addressed. However, its achievements regarding national implementation are significant, and may provide lessons for other countries.

## Sources of financial support

This work was funded by the Health Priorities and Suicide Prevention Branch of the Australian Government's Department of Health and Ageing.
